# Sequential intravital imaging reveals in vivo dynamics of pancreatic tissue transplanted under the kidney capsule in mice

**DOI:** 10.1007/s00125-016-4049-6

**Published:** 2016-07-21

**Authors:** Léon van Gurp, Cindy J. M. Loomans, Pim P. van Krieken, Gitanjali Dharmadhikari, Erik Jansen, Femke C. A. S. Ringnalda, Evelyne Beerling, Jacco van Rheenen, Eelco J. P. de Koning

**Affiliations:** 1 Hubrecht Institute—KNAW and University Medical Center Utrecht, Uppsalalaan 8, 3584CT Utrecht, the Netherlands; 2 Department of Internal Medicine, Leiden University Medical Center, Leiden, the Netherlands; 3 The Rolf Luft Research Center for Diabetes and Endocrinology, Karolinska Institutet, Karolinska University Hospital, L1, Stockholm, Sweden; 4 Department of Tumor Immunology, Radboudumc, Nijmegen, the Netherlands; 5 Institute of Molecular Health Sciences, ETH Zürich, Zürich, Switzerland; 6 Cancer Genomics Center, Hubrecht Institute—KNAW and University Medical Center Utrecht, Utrecht, the Netherlands

**Keywords:** Development, Embryonic, Human, Imaging, Intravital, Islets, Microscopy, Mouse, Pancreas, Transplantation

## Abstract

**Aims/hypothesis:**

Dynamic processes in pancreatic tissue are difficult to study. We aimed to develop an intravital imaging method to longitudinally examine engraftment, vascularisation, expansion and differentiation in mature islets or embryonic pancreases transplanted under the kidney capsule.

**Methods:**

Isolated pancreatic islets from adult mice and murine embryonic day (E)12.5 pancreases containing fluorescent biomarkers were transplanted under the kidney capsule of immunodeficient recipient mice. Human islet cells were dispersed, transduced with a lentivirus expressing a fluorescent label and reaggregated before transplantation. Graft-containing kidneys were positioned subcutaneously and an imaging window was fitted into the skin on top of the kidney. Intravital imaging using multiphoton microscopy was performed for up to 2 weeks. Volumes of fluorescently labelled cells were determined as a measure of development and survival.

**Results:**

Transplanted islets and embryonic pancreases showed good engraftment and remained viable. Engraftment and vascularisation could be longitudinally examined in murine and human islet cells. Murine islet beta cell volume was unchanged over time. Transplanted embryonic pancreases increased to up to 6.1 times of their original volume and beta cell volume increased 90 times during 2 weeks.

**Conclusions/interpretation:**

This method allows for repeated intravital imaging of grafts containing various sources of pancreatic tissue transplanted under the kidney capsule. Using fluorescent markers, dynamic information concerning engraftment or differentiation can be visualised and measured.

**Electronic supplementary material:**

The online version of this article (doi:10.1007/s00125-016-4049-6) contains peer-reviewed but unedited supplementary material, which is available to authorised users.

## Introduction

Islet cell development, dynamic processes involved in islet engraftment and changes in islet composition are difficult processes to study in vivo. To obtain longitudinal information in combination with functional data, new imaging methods are required that allow sequential measurements in individual animals.

Transplantation of pancreatic islets under the kidney capsule is considered the gold standard for the in vivo evaluation of graft insulin secretory capacity and survival in mice [1]. Isolated islets derived from humans and different animal species can be used for transplantation at this site [2]. After engraftment, there is good vascularisation of the transplanted islets, allowing the rapid release and action of insulin. Nephrectomy of the graft-containing kidney followed by determination of blood glucose values validates graft function [3]. By transplanting a cell pellet that remains compact after transplantation, grafts can easily be retrieved and histologically analysed.

In this study, we adapted our previously published method [4, 5] to perform intravital imaging on islets of Langerhans or embryonic pancreatic tissue transplanted under the kidney capsule using an abdominal imaging window. This method allows sequential measurements of survival, vascularisation, expansion and differentiation of these tissues for a period up to 2 weeks.

## Methods

### Mouse pancreatic islets and embryonic pancreas isolation

All experiments on animals were carried out in accordance with the guidelines of the Animal Welfare Committee of the Royal Netherlands Academy of Arts and Sciences. Mature pancreatic islets and intact E12.5 embryonic pancreases were isolated from mouse insulin promoter–enhanced green fluorescent protein (MIP-EGFP) (Jackson Laboratory, Bar Harbor, ME, USA, stock no. 006864) and cytomegalovirus–actin–globin promoter red fluorescent protein (CAG-DsRed) (Jackson Laboratory, stock no. 005441) mice. See ESM for further details.

### Human pancreatic islets and lentiviral transduction

Human pancreatic islets were isolated at the Leiden University Medical Centre using standard procedures [6]. Isolated human islets could only be used with research consent and when the number and/or quality of the islets were insufficient for clinical islet transplantation, according to national laws. Islets were transduced with a human insulin promoter–GFP (HIP-GFP) virus before transplantation. See ESM for further details.

### Islet transplantation and positioning of an intra-abdominal imaging window

NOD severe combined immunodeficiency gamma (NSG) mice (Jackson Laboratory, stock no. 005557) were anaesthetised and pancreatic islets or embryonic pancreases were transplanted under the kidney capsule. The kidney was fixed subcutaneously and an imaging window was placed in the skin (ESM Fig. 1a–f), as published previously [4]. See ESM for further details.

### Intravital imaging

Intravital imaging was performed as described previously [5]. If visualisation of the vasculature was required, mice received an intravenous tail vein injection of 2.5 mg 70 kDa dextran-Texas Red conjugate (Thermo Fisher, Waltham, MA, USA, D-1830) in 100 μl PBS directly before imaging. Images were acquired at resolutions between 0.6 × 0.6 × 1.0 and 4.8 × 4.8 × 5.0 μm (*xyz*). Multiphoton excitation was performed at 960 nm, and the emission was collected at 505–555 nm (EGFP), and 555–695 nm (DsRed, Texas Red).

### Histological analysis

Graft-containing kidneys were either fixed for paraffin sectioning or for cryosectioning. Paraffin-embedded sections (4 μm) were cut at and stained with haematoxylin and eosin for the determination of graft size and capsule thickness. Cryosections (10 μm) were used for immunohistochemical staining for insulin and glucagon. See ESM for further details.

### Microscopy data processing

See ESM for further details.

### Statistical analysis

Data are presented as mean ± SEM. R (www.R-project.org) was used to perform statistical analyses. IPGTT data were analysed using two-way ANOVA. All other statistical analyses were performed using independent two-way Student’s *t*-tests. Data were considered significant if the *p* value was <0.05.

## Results

### Transplanted islets function normally in mice fitted with an abdominal imaging window

Mice recovered quickly after surgery and were fully active after 1 h. There was no impairment of movement and mice did not show behaviour indicating pain or discomfort. Body weight increased normally after surgery (ESM Fig. 2a). To test if islet graft functionality was affected by the procedure, an IPGTT was performed and human C-peptide concentrations were measured in streptozotocin-induced hyperglycaemic NSG mice 4 weeks after transplantation of 2000 human islet equivalents. Both mice with an abdominal imaging window (AIW, *n* = 5) and controls (CTRL, *n* = 5) showed a normal response to an IPGTT (ESM Fig. 2b). Human C-peptide concentrations were similar between the two groups 4 weeks after surgery (358 ± 157 pmol/l [AIW] and 384 ± 47 pmol/l [CTRL], *p* = 0.88; ESM Fig. 2c). Graft thickness (801 ± 86 μm [AIW] and 827 ± 51 μm [CTRL]) was unaffected (*p* = 0.81). Capsule thickness was not significantly increased (477 ± 64 μm [AIW] and 324 ± 34 μm [CTRL], *p* = 0.09; ESM Fig. 2d). The imaging window did not affect the proportion of beta cells (relative to the total alpha and beta cells) in the transplanted islets (ESM Fig. 2e–g).

### Transplanted islets can be repeatedly monitored during in vivo engraftment

**Fig. 1:**
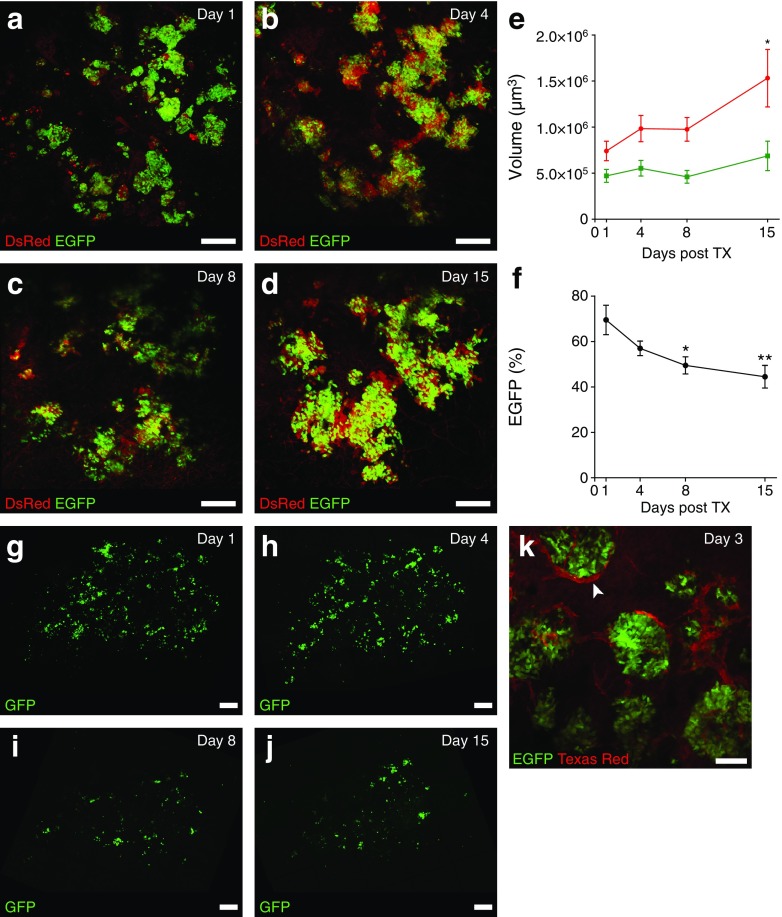
Vascularisation and survival of pancreatic islets transplanted under the kidney capsule of immune deficient NSG mice. (**a**–**d**) Maximum projection image (projecting all *xy* focal planes into a single 2D image) of murine MIP-EGFP/CAG-DsRed islets transplanted under the kidney capsule. The grafts were imaged on day 1, 4, 8 and 15. MIP-EGFP (green), CAG-DsRed (red). Scale bar, 250 μm. (**e**) Tissue volume of all pancreatic cells (CAG-DsRed, in red) and beta cells (MIP-EGFP, in green) in transplanted MIP-EGFP/CAG-DsRed islets over time. DsRed volume was significantly increased on day 15 compared with day 1. (**f**) Percentage of the volume of beta cells (EGFP %) over whole tissue volume (DsRed) in transplanted MIP-EGFP/CAG-DsRed islets over time. The percentage of beta cells was significantly decreased on day 8 and 15 compared to day 1. (**g**–**j**) Human pancreatic islets were dispersed into single cells, transduced with a lentivirus containing a HIP-GFP virus, and reaggregated overnight on ultra-low attachment plates. After 1 week in culture, islet cell aggregates were visually assessed and transplanted if at least 50% of the cells were fluorescent. Maximum projection images of transplanted islets were captured on day 1, 4, 8 and 15. HIP-GFP (green). Scale bar, 250 μm. (**k**) Image of a single *xy* focal plane of transplanted MIP-EGFP islets 3 days after transplantation. Blood vessels (red) were visualised after a tail vein injection of Texas Red-conjugated dextran solution. The arrowhead marks a transplanted islet of which several *xy* focal planes are merged into a mosaic in ESM Fig. 4. Scale bar, 100 μm. Data are mean ± SEM. *p < 0.05, **p < 0.01 vs day 1. TX, transplant

To demonstrate that longitudinal intravital imaging of tissue grafts under the kidney capsule can be performed, normoglycaemic NSG mice (*n* = 4) were transplanted with 10–25 mouse MIP-EGFP/CAG-DsRed islets and fitted with an AIW. Imaging was performed on day 1, day 4, day 8 and day 15 after surgery (Fig. 1a–d). In total, 29 separate islet-containing regions of interest were identified. In these areas, beta cell and total cell volume were measured by volumetric calculation of the DsRed and EGFP signal. DsRed volume at 15 days was 2.1 times that at day 1 (7.4 × 10^5^ ± 1.1 × 10^5^ μm^3^ at day 1 vs 15.3 × 10^5^ ± 3.1 × 10^5^ μm^3^ at day 15, *p* = 0.029), while EGFP volume did not significantly increase over this time (4.7 × 10^5^ ± 0.7 × 10^5^ μm^3^ at day 1 vs 6.9 × 10^5^ ± 1.6 × 10^5^ μm^3^ at day 15, *p* = 0.22) (Fig. 1e). The relative EGFP content decreased from 69.5 ± 6.5% on day 1 to 44.5 ± 5.0% on day 15 (*p* = 0.004) (Fig. 1f). To show that human islet cells can also be sequentially imaged, HIP-GFP-transduced human islet cell aggregates were transplanted under the kidney capsule. Approximately 50% of all transplanted cells expressed GFP after transduction prior to transplantation (ESM Fig. 3). Beta cells expressing GFP could be clearly visualised on days 1, 4, 8 and 15 after transplantation under the kidney capsule (Fig. 1g–j).

### Visualisation of islet graft vasculature and blood flow

Blood vessels were visualised in MIP-EGFP islet grafts after injection of Texas Red-labelled dextrans into the tail vein. The first signs of vascularisation were recorded 3 days after transplantation, when blood vessels could be identified within and surrounding the islets (Fig. 1k, ESM Fig. 4).

### Transplanted embryonic pancreas increases in size and forms islets within 2 weeks

**Fig. 2:**
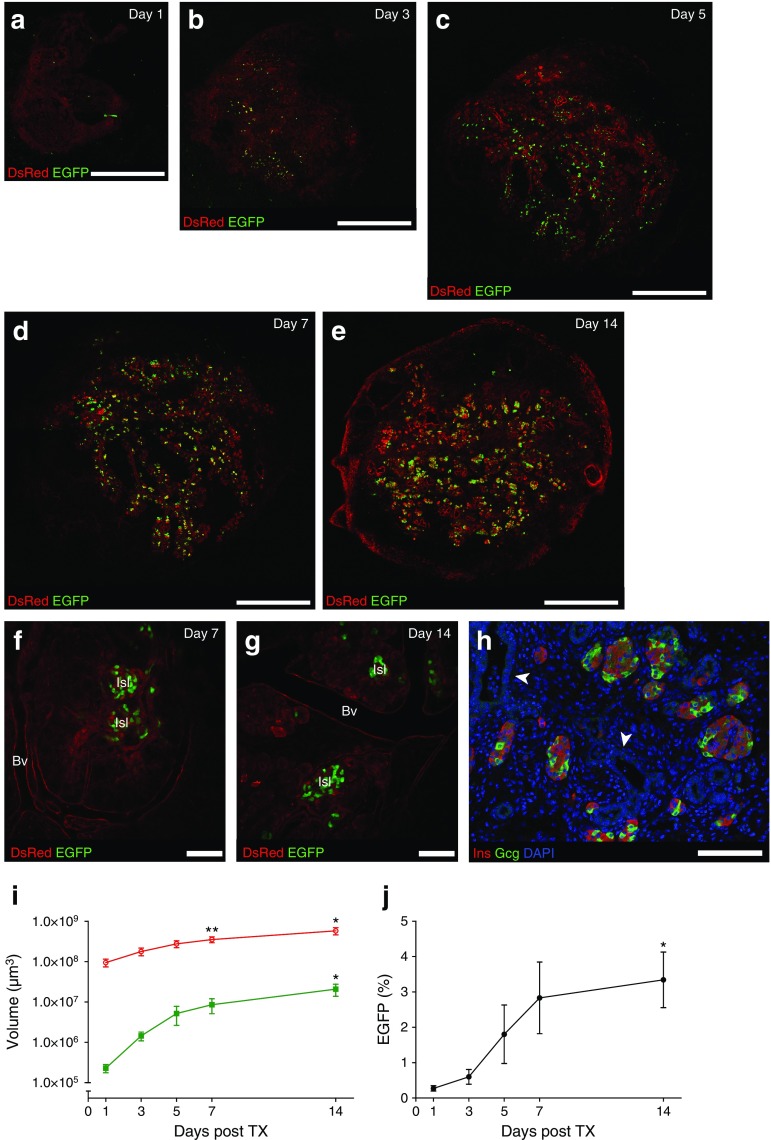
Expansion and differentiation of MIP-EGP/CAG-DsRed embryonic pancreas (E12.5) under the kidney capsule of immunodeficient NSG mice. (**a**–**e**) Images of individual *xy* focal planes of embryonic pancreases from MIP-EGFP/CAG-DsRed mice transplanted under the kidney capsule and imaged on day 1, 3, 5, 7 and 14. MIP-EGFP (green), CAG-DsRed (red). Scale bar, 1000 μm. (**f**–**g**) Higher magnification of individual *xy* focal planes showing islets in the transplanted embryonic pancreas (red) on day 7 and 14 after transplantation. Blood vessels (Bv) and islets (Isl) can be clearly distinguished. MIP-EGFP (green), CAG-DsRed (red). Scale bar, 250 μm. (**h**) Immunohistochemical staining for insulin (red), glucagon (green) and DAPI (blue) 14 days after transplantation of embryonic pancreatic tissue. Arrowheads mark ductal structures. Scale bar, 250 μm. (**i**) Volume of the total embryonic pancreatic tissue (CAG-DsRed, in red) and the insulin-expressing cells (MIP-EGFP, in green) after transplantation. DsRed volume was significantly increased on day 7 and 14 compared with day 1, and EGFP volume was significantly increased on day 14 compared with day 1. (**j**) Percentage of the volume of the insulin-expressing cells (EGFP %) out of the total embryonic pancreatic tissue volume (DsRed) after transplantation. EGFP volume was significantly increased on day 14 compared with day 1. Data are mean ± SEM. *p < 0.05 and **p < 0.01 vs day 1. TX, transplant


To demonstrate that large tissue grafts can be visualised and to monitor pancreatic development in a quantitative fashion, we transplanted two to four E12.5 embryonic pancreases from pregnant MIP-EGFP/CAG-DsRed mice under the kidney capsule of NSG mice before an AIW was fitted (*n* = 7). One day after transplantation, 27.3 ± 3.6 EGFP-positive cells were present per embryonic pancreas (*n* = 18). These cells were usually single and dispersed throughout the embryonic pancreas (Fig. 2a). When multiple pancreases were transplanted, the grafts joined together over time (Fig. 2a–e). After 7 days, the first islet-like clusters were visible in the grafts (Fig. 2f, ESM movie). Mature islets were present 2 weeks after transplantation, as illustrated by rounded and separated EGFP-positive cell clusters during intravital imaging (Fig. 2g) and immunohistochemical staining for insulin and glucagon (Fig. 2h). DsRed volume at 14 days was 6.1 times that at day 1 (9.5 ± 2.1 × 10^7^ μm^3^ at day 1 vs 5.8 ± 1.2 × 10^8^ μm^3^ at day 14, *p* = 0.014), and EGFP volume was 90 times that at day 1 (2.3 ± 0.5 × 10^5^ μm^3^ at day 1 vs 2.1 ± 0.7 × 10^7^ μm^3^ day 14, *p* = 0.04) (Fig. 2i). After 2 weeks, the EGFP-positive cells represented 3.34 ± 0.78% of the DsRed-positive cells (Fig. 2j).

ESM movieLive intravital imaging movie of E12.5 MIP-EGFP::CAG-DsRed embryonic, 7 days after transplantation under the kidney capsule. Functional blood vessels are visualized in red, and erythrocytes can be observed moving through them. MIP-EGFP (green), CAG-DsRed (red). MIP-EGFP: Enhanced green fluorescent protein (GFP) under the transcriptional regulation of the mouse insulin promoter (MIP). CAG-DsRed: Red fluorescent protein (DsRed) under the transcriptional regulation of the cytomegalovirus-actin-globin (CAG) promoter. (MP4 26101 kb)


## Discussion

Here we describe a novel intravital imaging model to study mature islet cells and developing pancreatic tissue using an abdominal imaging window. Tissues transplanted under the kidney capsule, which is considered the gold standard site for studying islet grafts, can be sequentially imaged for at least 2 weeks. Using fluorescent markers, we longitudinally measured engraftment, expansion, differentiation and vascularisation in transplanted developing pancreatic tissue and mature islets.

Alternative intravital imaging methods have been used to study islet function, survival and vascularisation over time. While the anterior chamber of the eye model allows prolonged (up to 6 months) sequential intravital imaging of transplanted pancreatic islets [7], it is limited by the tissue volume that can be transplanted. This reduces options to transplant large amounts of tissue, or tissues with expansion capacity over time.

Our model allows the dynamic characterisation of the embryonic pancreas, which undergoes further development and expansion after transplantation under the kidney capsule [8]. Fetal pancreases are by nature highly proliferative [9]. The time course of growth and differentiation of embryonic pancreases transplanted under the kidney capsule is very similar to that observed in eutopic pancreatic development [10]. Sequential intravital imaging shows the process of islet neogenesis, as indicated by the appearance of rounded clusters of fluorescent cells. In the majority of embryonic pancreatic tissue transplants no acinar tissue was observed, as reported previously [8]. This is probably the reason why the percentage of beta cells in our grafts is relatively high compared with normal pancreatic development.

We were also able to dynamically assess transplanted islet grafts. Islets were connected to the host vasculature within 3 days, which is in line with previous findings [7]. Generally, placement of an imaging window did not affect survival or function of islets transplanted under the kidney capsule.

Important processes such as engraftment, vascularisation, expansion and differentiation can be studied using this imaging method. The technique can therefore be a valuable tool in beta cell replacement therapy using progenitor cells, islet inflammation and rejection. In conclusion, we have developed a novel method to dynamically image both mature islet cells and developing pancreatic tissue.

## Electronic supplementary material

Below is the link to the electronic supplementary material.

ESM (PDF 24084 kb)

## Abbreviations

AIW: Abdominal imaging window
CAG-DsRed: Cytomegalovirus–actin–globin promoter red fluorescent protein
GFP: Green fluorescent protein
HIP-GFP: Human insulin promoter-green fluorescent protein
MIP-EGFP: Mouse insulin promoter-enhanced green fluorescent protein
NSG mice: NOD severe combined immunodeficiency gamma mice
