# Supplementary material for: Sequential intravital imaging reveals in vivo dynamics of pancreatic tissue transplanted under the kidney capsule in mice
Source: Diabetologia. 2016 Jul 21;59(11):2387–92. doi: 10.1007/s00125-016-4049-6 (PMC5506096; doi:10.1007/s00125-016-4049-6)
Supplement: Supplementary file 2 — (PDF 24084 kb) [file 125_2016_4049_MOESM1_ESM.pdf]

Supplementary Methods:

Mouse pancreatic islet isolation Animals between 7 and 12 weeks of age were killed after which pancreases were dissected from the animals. Pancreatic tissue was then dissected into small pieces (approximately 1 mm^3). These pieces were incubated in 3ml RPMI medium (Thermo Fisher, 72400-021) containing 3mg/ml collagenase (Sigma Aldrich, St. Louis, MO, USA, C9263) in a shaking incubator at 37°C until a homogenous digest was obtained. Islets were immediately washed in RPMI medium containing 10% FBS, handpicked based on EGFP expression and transplanted within 4 hours after isolation.

Mouse embryonic pancreas isolation Embryonic pancreases were dissected from E12.5 mouse embryos as described before [1] and kept in BME medium (Thermo Fisher, 4101-026) containing 100U/ml penicillin-streptomycin (Thermo Fisher, 15140-122), 50µg/ml gentamicin (Thermo Fisher, 15710-049), 1% glutamax (Thermo Fisher, 35050-061) and 10% FBS on ice until transplantation.

Human pancreatic islets and lentiviral transduction For lentiviral transduction, the human islets were first dispersed into single cells using TrypLE (Thermo Fisher, 12605-010) containing 10µg/ml pulmozyme (Roche, Basel, Switzerland). A lentivirus containing a green fluorescent protein (GFP) under transcriptional regulation of the human insulin promoter (HIP) [2] was used. The HIP-GFP lentivirus was added to the dispersed cells at 20µg/ml, centrifuged at 300*g* for 1 hour, followed by incubation at 37°C for 3 hours. Cells were washed in PBS and cultured in RPMI medium containing 10% FBS on ultra-low attachment plates (Corning, Corning, NY, USA, 3471) during which spontaneous reaggregation occurred.

Islet or embryonic pancreas transplantation and positioning of an intra-abdominal imaging window NSG mice were anesthetized using isoflurane. The lateral abdominal area on the left flank was shaved and an incision was made in both skin and abdominal muscle over the left kidney. The kidney was exposed and either 10-25 mouse islets, 2000 human islet equivalents (IEQ) or 2-4 embryonic pancreases were transplanted under the kidney capsule. A purse-string suture was made in the abdominal muscle and the kidney was fixed in place by tightening the suture which prevented the kidney from recoiling into the abdominal cavity, while blood vessels and ureter remained intact. A second purse-string suture was made in the skin and an abdominal imaging window (AIW) [3] was fitted into the incision. Tightening the suture fixed the window in the skin, leaving the graft directly underneath it (sFig 1). Mice received a single dose of 3µg buprenorphin (Temgesic, RB Pharmaceuticals Ltd, Richmond, VA, USA) as

post-surgical pain relief. In control animals, no AIW was fitted, and the kidney was returned to the retroperitoneal space before muscle and skin closure using standard procedures.

Histological analysis After killing the mice, the graft-containing kidney was removed and either fixed overnight in a 4% paraformaldehyde solution and embedded in paraffin, or fixed overnight in 1% paraformaldehyde solution in PBS containing 2.12mg/ml sodium periodate and 75mM l-lysine, cryoprotected in 30% sucrose for 6 hours and frozen in tissue freezing medium (Leica, Wetzlar, Germany, #14020108926). Paraffin sections were cut at 4µm and stained with haematoxylin and eosin for the determination of graft size and capsule thickness. Six to nine haematoxylin and eosin stained tissue sections per graft were analysed with sections being 100-150µm apart. The measurement in every section comprised the largest diameter of the graft and capsule using Leica LAS AF 2.6 software. A mean diameter for graft and capsule per mouse was calculated. Cryosections were used for immunohistochemical staining. Primary antibodies against insulin (1:100, Ab7842, Abcam, Cambridge, UK) and glucagon (1:100, VP-G806, Vector labs, Burlingame, CA, USA) were used. Biotin-conjugated donkey anti rabbit (1:200, 711-065-152, Jackson Immunoresearch Ltd, West Grove, PA, USA), TRITC anti guinea-pig (1:400, 706-025-148, Jackson Immunoresearch Ltd) and Alexa 488 streptavidin (1:200, S-11223, Life Technologies) were used as secondary antibodies. Bright field images were acquired using a DM4000 microscope (Leica) with LAS AF software version 3.1. Fluorescent images were acquired using an inverted TCS SP5 microscope (Leica) with a 4W multiphoton chameleon Ti:Sapphire–pumped optical parametric oscillator (Coherent Inc, Santa Clara, CA, USA). For DAPI, multiphoton excitation was performed at 780nm. Other fluorophores were excited using single photon laser lines. Alexa488 and EGFP were excited at 488nm, and Alexa568, TRITC and DsRed at 561nm. Emission was collected at 400-460nm for DAPI, 510-550nm for Alexa488 and EGFP, and 570-610nm for Alexa568, TRITC and DsRed.

Microscopy data processing Image stacks from grafts that covered multiple fields of view were stitched together and exported using LAS AF software 2.6 (Leica). Maximum projection images were generated using ImageJ 1.49k. Reconstructions in 3D and volumetric calculations were made using Volocity software version 6.0 (PerkinElmer, Waltham, MA, USA).

Supplementary methods references

[1] Petzold KM, Spagnoli FM (2012) A system for ex vivo culturing of embryonic pancreas. Journal of visualized experiments : JoVE: e3979

[2] Zaldumbide A, Alkemade G, Carlotti F, et al. (2013) Genetically engineered human islets protected from CD8-mediated autoimmune destruction in vivo. Molecular therapy : the journal of the American Society of Gene Therapy 21: 1592-1601

[3] Ritsma L, Steller EJ, Ellenbroek SI, Kranenburg O, Borel Rinkes IH, van Rheenen J (2013) Surgical implantation of an abdominal imaging window for intravital microscopy. Nature protocols 8: 583-594

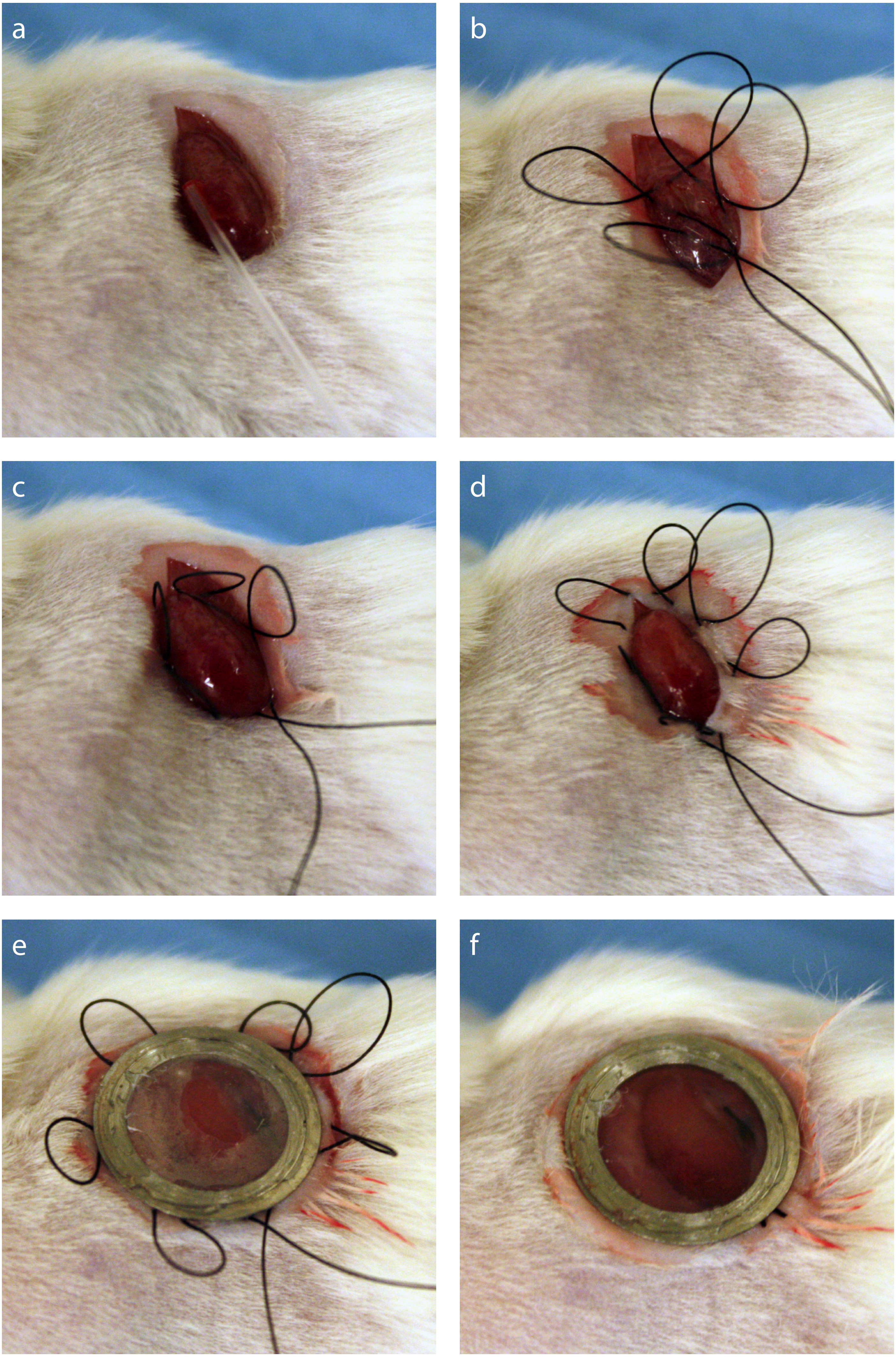

ESM FIGURE 1

ESM figure 1 Procedures for abdominal window insertion [4] and intravital microscopy of grafts transplanted under the kidney capsule. (**a**) The skin and abdominal muscle of the left flank are opened, the kidney is surfaced and pancreatic islets or embryonic pancreases are transplanted using standard procedures. (**b**) The kidney is replaced into the retroperitoneal space and a purse-string suture is made in the abdominal wall. (**c**) The kidney is repositioned outside the abdominal wall and the purse-string suture is tightened so that the kidney is now positioned in the subcutaneous space. (**d**) A similar purse-string suture is made into the skin surrounding the incision. (**e**) An abdominal imaging window is placed into the incision in the skin. (**f**) Tightening the purse-string suture in the skin fixes the window in place, creating an airtight barrier.

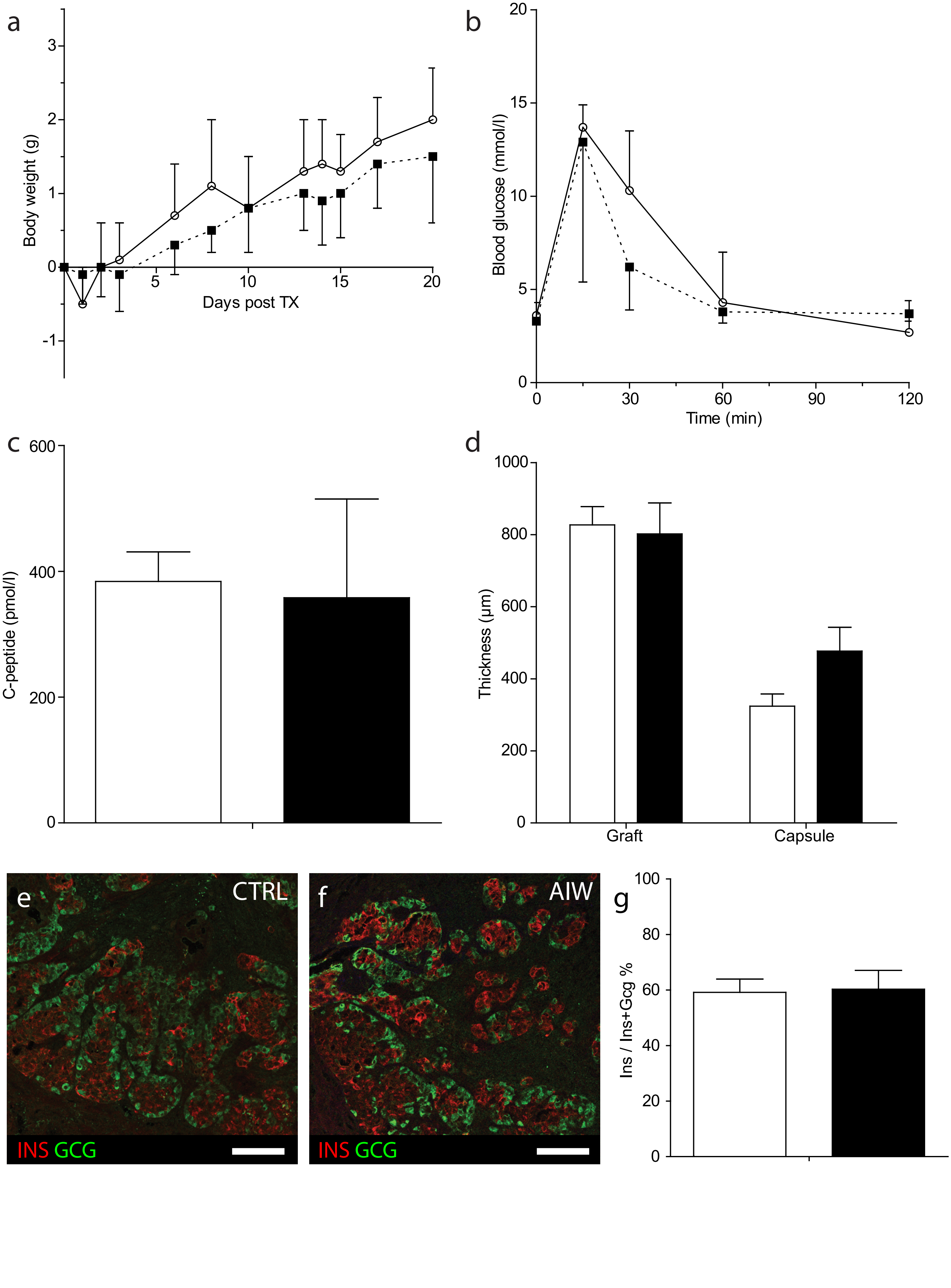
a
Body weight (g)
Days post TX
b
Blood glucose (mmol/l)
Time (min)
c
C-peptide (pmol/l)
d
Thickness (µm)
Graft
Capsule
e
CTRL
INS GCG
f
AIW
INS GCG
g
Ins / Ins+Gcg %

ESM FIGURE 2

ESM figure 2 Effects of an abdominal imaging window insertion. NSG mice were treated with a single intraperitoneal injection of 130 mg/kg streptozotocin (Sigma Aldrich, S0130) to induce hyperglycemia. Insulin glargine (Lantus, Sanofi-Aventis) was administered subcutaneously if blood glucose concentrations were >20mmol/l. (**a**) Change in body weight of mice with (AIW) or without (CTRL) an abdominal imaging window after transplantation of 2000 IEQ human islets (n=5 per group). (**b**) An IPGTT was performed on AIW and CTRL mice that were fasted for 16 hours, 28 days after surgery. Baseline blood glucose was measured, after which mice received a 3 mg/kg intraperitoneal injection of glucose. Blood glucose concentrations were determined at 15, 30, 60 and 120 minutes post injection. (**c**) Human C-peptide concentrations on day 28 after surgery in AIW and CTRL NSG mice transplanted with human islets. (**d**) Islet graft size and fibrous capsule thickness. (**e-f**) Immunohistochemical staining for insulin and glucagon in sections from human islet grafts 28 days after transplantation. (**g**) Percentage of insulin positive cells with respect to all insulin and glucagon positive cells. CTRL = lines & open circles, or white bars. AIW = dotted lines & closed squares, or black bars. Data are mean ± SEM. Scale bar 250 µm.

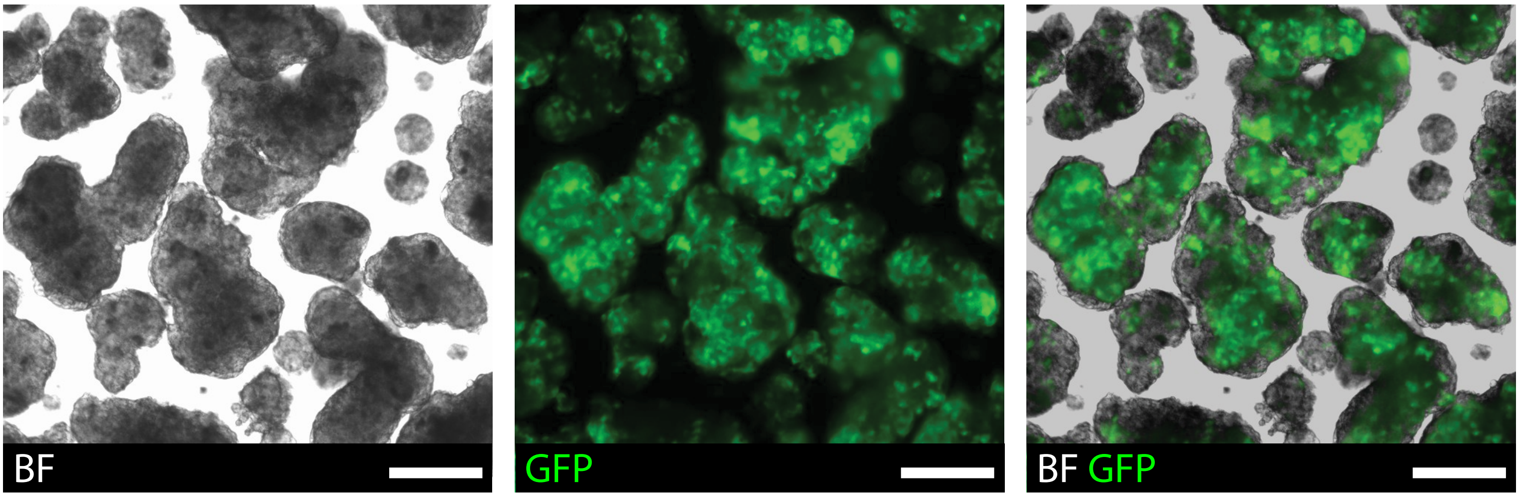

ESM FIGURE 3

ESM figure 3 Transduction efficiency of human islet aggregates after a single-cell transduction with a HIP-GFP virus, reaggregation and culture for 7 days. HIP-GFP: Green fluorescent protein (GFP) under the transcriptional regulation of the human insulin promoter (HIP). Scale bar 250 µm.

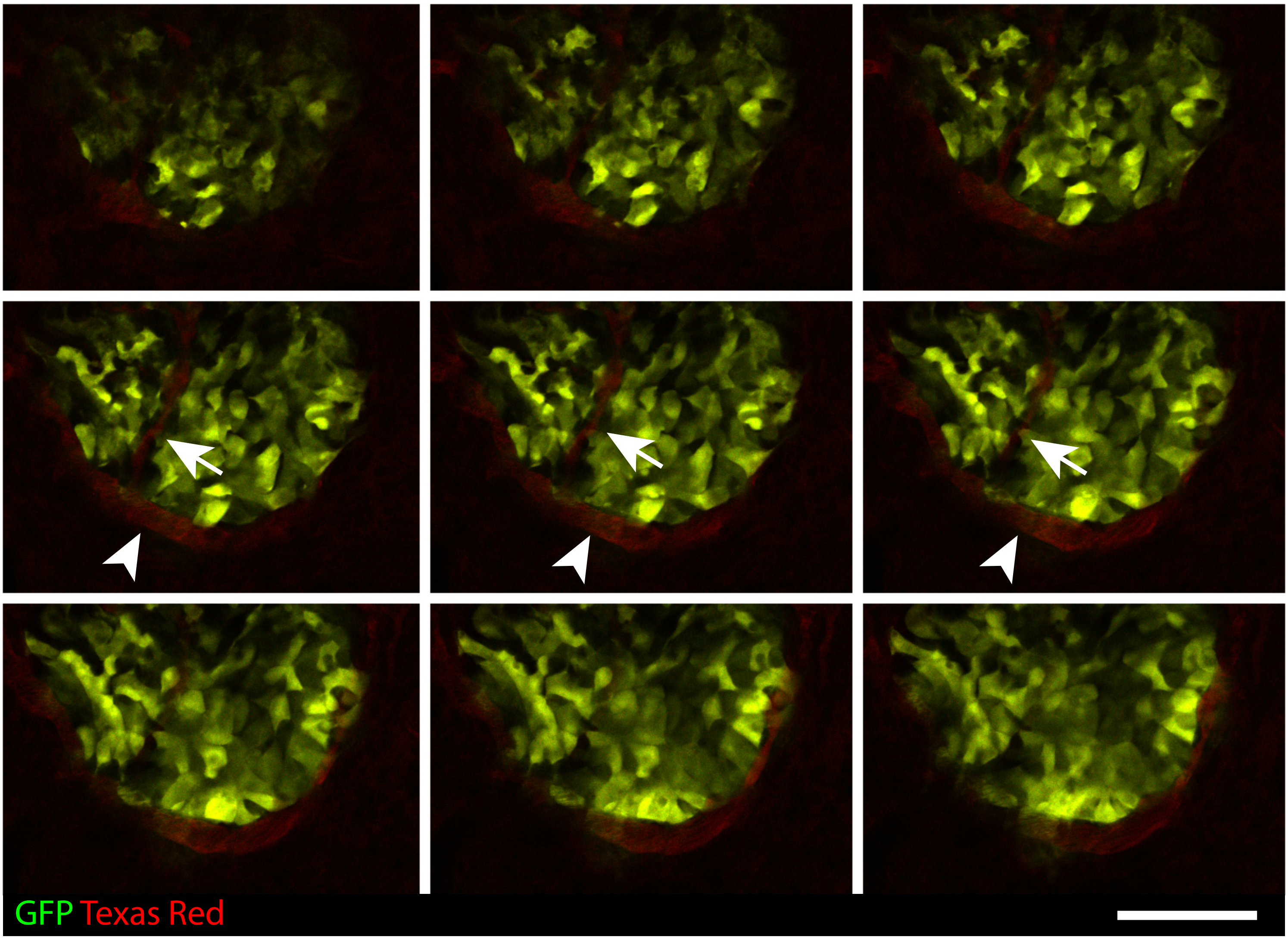

ESM FIGURE 4

ESM figure 4 Mosaic of individual XY focal plane images of MIP-EGFP islets three days after transplantation, and Texas Red labelled blood vessels inside these islets. The distance between individual images is 2µm. Arrowheads indicate a blood vessel surrounding the islet, arrows indicate a blood vessel inside the islet. Scale bar 100 µm.
